# Characteristics of Fungal Communities and Internal Mildew Occurrence during the Stages of Planting and Storing of Sunflower Seed in China

**DOI:** 10.3390/microorganisms10071434

**Published:** 2022-07-15

**Authors:** Jie Liu, Yang Yang, Zhuopin Xu, Qi Wang, Binmei Liu, Yuejin Wu

**Affiliations:** 1 Hefei Institutes of Physical Science, Chinese Academy of Sciences, Hefei 230031, China; jieliu1107@163.com (J.L.); yangyang19860207@163.com (Y.Y.); xuzp@iim.ac.cn (Z.X.); wangqi@ipp.ac.cn (Q.W.); 2 Science IsIand Branch, Graduate School of USTC, Hefei 230026, China

**Keywords:** sunflower seeds, internal mildew, *Alternaria*, high-throughput sequencing

## Abstract

Internally mildewed sunflower seeds pose a significant risk to human health. To control internal mildew, it is imperative to study its source in the main production area of China, which has been little investigated. Here, high-throughput sequencing was used to characterize the fungal and fungus-seed communities. Alpha diversity and ANOSIM analyses showed mildew did not alter the fungal compositions significantly. STAMP analysis showed that the sunflower seeds were most vulnerable to internal mildew during the field-planting stage. *Alternaria* was the predominant mildew-causing pathogen of sunflower seeds for consumption, which may originate from seed transmission and colonize at the seed-development stage. Finally, only a few seeds developed internal mildew with a worrisome level of *Alternaria* contamination in the humid field climate. NMDS analysis showed that climatic factors also played important roles in shaping microbial change during storage, with a relative humidity (RH) of 67% being the critical threshold in normal-temperature warehouses. Internal mildew never occurred below the RH threshold for the microbial community structure, which hardly changed after an average storage duration. The results indicated that a combination of field management to combat *Alternaria*, pretreatment with 5 KGy γ-irradiation and drying at the time of storage will minimize or prevent internal mildew. This work also provides an empirical framework for studies of mildewing in other shelled seeds.

## 1. Introduction

Sunflowers are widespread and suitable for agriculture in all regions of the world. China is the fourth largest producer of sunflower seeds worldwide after Ukraine, Russia and Argentina [1]. Sunflower seeds and oil contain unsaturated fatty acids, proteins, human-essential amino acids, fiber, and vitamins [2]. With the global increase in public health awareness, consumers increasingly make healthy choices based on ingredients. Sunflower seeds and related foods will benefit from this increased demand, leading to enhanced interest from consumers and producers. 

One of the main problems facing sunflower seed production is internal mildew, meaning that the kernels become mildewed or discolored inside a normal shell [3]; internal mildew may cause a number of severe toxic effects in humans [4,5,6]. In China, sunflower seeds are typically stored, transported, and marketed as whole seeds with the shell intact, and shelled sunflower seeds are usually eaten or used for the direct extraction of edible oil [7,8]. Because most internally moldy seeds have a normal-looking shell and do not show external evidence of mildew, these seeds cannot be recognized and discarded by the naked eye or color-sorting equipment. For this reason, accidental ingestion is frequent, negatively affecting human health, as well as leading to serious economic losses to producers and farmers.

Over recent decades, the risks associated with the contamination of agricultural products with mildew, especially grains, have received extensive research attention worldwide [9]. However, studies of the second important group of agricultural plants after grains, the oil-seeds, primarily represented by sunflowers, lag significantly behind the grains. This discrepancy must be overcome. Recent studies have been limited to characterizations of the microbial compositions of normal sunflower seeds from various regions, including Europe, South America, and Southeast Asia [2,10,11]. However, very few reports have investigated the relationship between microorganisms and internal mildew. Additionally, microbial community characterization requires an accurate detection method. The identification of sunflower-seed-associated fungal species has traditionally been performed using traditional isolation and culturing techniques [10,11,12,13]. As most microorganisms require specific culture conditions, the microorganisms isolated using the plate-culture method only account for 1–10% of the total [14]. In contrast, the high-throughput sequencing can qualitatively and quantitatively give precise information to identify the microbial community without culturing. Additionally, the high-throughput sequencing could also analyze the dynamic change of microbial communities. In addition, almost all previous studies have been restricted to characterizations of the microorganisms carried on the seed’s surface, disregarding the endophytic microorganisms. This oversight may have led to inaccurate conclusions, as most infecting fungi can penetrate into the kernel and multiply [15,16]. 

It is practical to avoid internal mildewing and the entry of potential mycotoxins into the food chain by controlling the growth of mildew-causing pathogens. To address this need, in this study, 18S high-throughput sequencing was used to precisely characterize the species community of sunflower-seed kernels, aiming to study the source of internal mildew in the main production area of China, as well as related influencing factors. This work also provides an empirical framework for studies of other shelled seeds, which may reduce the risk of human exposure to internal mildewing.

## 2. Materials and Methods

### 2.1. Material Collection and Sampling Strategy

Inner Mongolia is the largest sunflower production area in China, with the city Bayan Nur well-known as “the hometown of sunflowers in China”. Normal and internally moldy samples were obtained from a sunflower-seed factory in Bayan Nur, Inner Mongolia, both immediately after harvest (post-harvest) and after 6–8 months of storage (post-storage) in normal-temperature warehouses. Samples were obtained over three years (2018–2020). The internally moldy samples were identified and collected over several months by factory workers due to the low occurrence rate of internal mildew. In total, we collected 330 internally moldy post-storage seeds in 2018, 534 in 2019, and 467 in 2020; we collected 453 internally moldy post-harvest seeds in 2019, and 424 in 2020. 

Our sampling strategy was designed to guarantee a representative sample. Sunflower seeds provided by the factory were gathered throughout Inner Mongolia. Furthermore, all kernels in each sample were mixed and reduced to about 70 using the quarter method before DNA extraction [17]. All samples were immediately transported to the laboratory (at –20 °C) and analyzed. 

### 2.2. Microbial Changes and the Occurrence of Internal Mildew

#### 2.2.1. DNA Extraction and Sequencing

To simultaneously obtain both the exophytic and endophytic microorganisms, we used the homogenization method. The kernels were fragmented in liquid nitrogen and mixed well. Next, 3 g of the ground kernel was added to the lysis solution (Hipure Soil DNA Kit, Magen Biotech Corporation, Guangzhou, China) with glass beads of different sizes. The kernel tissue was then crushed using a tissue disrupter (Tissuelyser-24, Shanghai Jingxin, Shanghai, China) at an oscillation frequency of 30 Hz for 5 min to generate finely ground powder without obvious particles. DNA was isolated from the powdered tissue using a commercial DNA extraction kit (Hipure Soil DNA Kit, Magen Biotech Corporation, Guangzhou, China) and quantified using a Qubit 3.0 fluorometer (Invitrogen Corporation, Carlsbad, CA, USA). The quality of the extracted DNA was assessed using electrophoresis on a 2% agarose gel (biowest agArose, Barcelona, Spain). All extracted DNA samples were stored at −20 °C until further analysis.

Library preparation and Illumina MiSeq sequencing were performed by Genewiz, Inc. (Suzhou, China). The library sequences (the V7 and V8 hypervariable regions of 18S rRNA) were amplified using polymerase chain reactions (PCRs) on a GeneAmp 9700 (ABI, Waltham, MA, USA) with the forward primer 5′-CGWTAACGAACGAG-3′ and the reverse primer 5′-AICCATTCAATCGG-3′. Each PCR volume (25 µL) contained 2.5 µL of TransStart buffer, 2 µL of dNTPs, 1 µL of each primer, 0.5 µL of TransStart Taq DNA polymerase, and 20 ng of DNA template. The first-round PCR products were used as templates for a second round of PCR amplicon enrichment (3 min of denaturation at 94 °C; 24 cycles of 5 s of denaturation at 95 °C, 90 s of annealing at 57 °C, and 10 s of elongation at 72 °C; and a final extension at 72 °C for 5 min). Indexed adapters were added to the ends of the amplicons using PCR to generate indexed libraries for downstream next generation sequencing. The DNA libraries were purified using magnetic beads, validated using 1.5% agarose gel electrophoresis, and quantified using a microplate reader (Infinite 200 Pro, Tecan, Männedorf, Switzerland). Finally, next-generation sequencing with dual reads was performed by Genewiz, Inc. (South Plainfield, NJ, USA) on an Illumina MiSeq/Novaseq Platform (Illumina, San Diego, CA, USA), following the manufacturer’s instructions. The sequences generated in this study have been deposited in the Sequence Read Archive of the National Center for Biotechnology Information (https://www.ncbi.nlm.nih.gov/biosample) (accessed on 31 March 2022) under PRJNA770022.

#### 2.2.2. Statistical Analysis

After demultiplexing, we obtained raw paired-end reads for each sample. The QIIME (Quantitative Insights Into Microbial Ecology; ver. 1.9.1) [18] data analysis package was used to analyze the 18S rRNA data. The forward and reverse reads were truncated by removing the index and primer sequences. Reads were joined only when the overlap was at least 20 bp. Quality filtering of the joined sequences was performed, and sequences that did not fulfill the following criteria were discarded: sequence length > 200 bp, no ambiguous bases, and mean quality score ≥ 20. The remaining high-quality sequences were compared with a reference database [Ribosomal Database Project (RDP) Gold database, ver.2.2] [19] using the UCHIME algorithm to detect and remove chimeric sequences. Effective sequences were grouped into OTUs at 97% sequence using the clustering program VSEARCH (ver.1.9.6) [20] against Silva 138 database, which had been pre-clustered at 97% sequence identity. The RDP Classifier was used to assign taxonomic categories to all OTUs at confidence threshold of 0.8.

In the work, for studying the relationship between internal mildew and microbial changes, 18S high-throughput sequencing was adopted to acquire the sequence of fungus-seed symbionts (fungi across the kernels and the kernel itself). The relative abundance of microbial OTUs based on the number of sequence reads is proportional to the degree of microbial contamination. The abundance of specific mildew-causing pathogen OTUs can be used as a proxy for the incidence of mildew. Thus, this method can identify the contamination degree of mildew-causing pathogens, and further investigate the occurrence and development process of internal mildewing by quantitatively analyzing the similarities and differences among the fungus–seed symbionts. The fungal community structure, which could also be acquired, was insufficient to support it. However, in this work, the fungal community structure was also acquired to analyze the effects on the kernels after mildewing. Additionally, for developing appropriate controlling methods, important influencing factors promoting mold development need to be explored comprehensively. Biological factors such as insect infestations can also lead to fungal community changes and internal mildewing by increasing seed water content and local temperature [21,22]. It can also be identified by 18S throughput sequencing. Meanwhile, 18S high-throughput sequencing is more suitable for high-level classification (genus or above) for sequence conservation. The reliability of the sequencing data was tested using rarefaction curves, which were generated in QIIME and visualized using the R package [23].

Alpha diversity indexes were calculated in QIIME using the Chao1 and ACE indexes. An analysis of similarity (ANOSIM) was used to quantify differences in the fungal community composition between mildewed and normal sunflower-seed kernels. To identify the stage at which internal mildewing occurred and developed, differences between post-harvest and post-storage sunflower-seed kernels at the genus level were compared using STAMP (v2.1.3) quantitatively [24]. Probability (*p*) values of < 0.05 were considered statistically significant. In addition, FUNGuild was used to predict the functions of the fungal communities in sunflower-seed kernels [25].

### 2.3. Identification of Pathogen Causing Mildew 

#### 2.3.1. Fungal Isolation and Molecular Identification

The moldy post-storage sunflower seed kernels were crushed and mixed well in liquid nitrogen. About 2 g powder of each sample was mixed with 200 mL sterile water in a shaker bottle and incubated at 30 °C for 20 min with shaking at 200 rpm. Next, the mixture was centrifuged at 1000 g for 10 min, and an aliquot of the upper solution was diluted to final concentrations of 10^−1^, 10^−2^, and 10^−3^ CFUs/mL. Each dilution was plated on the separation medium: high-salt Rose Bengal medium supplemented with 3 g/L sodium chloride; this separation medium reduces the growth of filamentous fungi and isolates the fungi. Sterile water was plated simultaneously as a control. After culture for 7 days in the dark at 28 °C, single colonies with the same morphology were selected from the mixed colonies and purified repeatedly with purification medium (potato dextrose agar, PDA). The final purified strains were transferred to purification medium and stored at 4 °C.

Fungal isolates were identified using molecular analysis. The mycelia from each pure fungal isolate were placed in a sterilized mortar, immediately frozen in liquid nitrogen, and ground into dry powder. Genomic DNA was extracted from powder using a fungal genomic DNA isolation kit (Sangon Biotech Co., Ltd., Shanghai, China). We then used PCR to amplify the nuclear ribosomal DNA ITS region using the primers ITS1(5′-TCCGTAGGTGAACCTGCGG-3′) and ITS4 (5′-TCCTCCGCTTATTGATATGC-3′). Each PCR mixture (25 µL) contained 0.5 µL of genomic DNA (20–50 ng/µL), 0.5 µL of each primer (10 µM), 0.2 µL of Taq PCR Master mix, 2.5 µL of 10× PCR buffer, and 1 µL of dNTPs (2.5 mM) in sterile deionized water. The PCR cycling conditions were as follows: 94 °C for 4 min; 30 cycles of 94 °C for 45 s, 55 °C for 45 s, and 72 °C for 1 min; and a final extension of 72 °C for 10 min. The PCR products were sequenced by Sangon Biotech (Shanghai) Co., Ltd. (Shanghai, China) using a 2720 thermal cycler (Applied Biosystems, Foster City, CA, USA). The obtained ITS sequences were searched against the NCBI GenBank database using BLAST [26].

#### 2.3.2. Pathogenicity Identification

To confirm the pathogenicity of the isolated fungi, pathogenicity testing of the fungal isolates was performed according to Koch’s postulates. Isolates of the most abundant fungus (as determined via sequencing and statistical analysis) were gently scraped from purification medium and mixed with sterile water to produce a suspension of 1 × 10^5^ cells/mL. Then, the healthy sunflower seeds were inoculated with this suspension, while the uninfected kernels were treated with sterilized water as a blank control. All treatments were performed in triplicate. Treated kernels were placed in Petri dishes and sealed with film to maintain suitable relative humidity (RH). The sealed Petri dishes were incubated in a biochemical incubator (SHP-300, Shanghai Sanfa Scientific Instruments Co., Shanghai, China) in the dark at 28 °C for 7 days. During incubation, the samples were inspected every day until the 7th for signs of mold. Once mildew appeared, the fungi were again isolated from the mildewed kernel and identified based on ITS sequence to determine if the mold fungus was the same species as was initially infected.

### 2.4. The Conditions Required for Internal Mildewing during Storage 

In preliminary experiments, we found that kernels with different initial moisture levels rapidly reached similar moisture levels under the same RH. Thus, post-harvest, unmildewed sunflower seeds were allowed to sit at different RHs under natural storage temperatures in the primary production area for six months to determine the critical threshold RH. First, saturated solutions of five different salts (KCl, NaCl, NH_4_NO_3_, NaBr, and K_2_CO_3_) [27] were placed in desiccators (Jiangsu Huaou Corp., Yancheng City, China) to generate environments with an RH of 84, 75, 67, 57, or 43%, respectively. When the RH in each desiccator was fully equilibrated, 200 g of post-harvest, unmildewed sunflower seeds were sealed in separate desiccators and incubated. After six months, the seeds were dehulled, and the kernels were examined. The moisture content of the kernels was measured using the direct drying method in the 1996 International Seed Inspection Regulations [28], and microbial community structure was assessed using high-throughput sequencing as described above [2.2.1]. We also analyzed β-diversity using NMDS to investigate microbial community similarities among kernels stored at different RHs, based on weighted and unweighted UniFrac distances [23]. We considered stress values ≤0.2 to indicate reliable results. The samples stored under different RHs were compared with normal post-harvest samples.

### 2.5. Irradiation Treatment to Prevent the Occurrence of Internal Mildewing during Storage

#### 2.5.1. γ-ray Irradiation

Unmildewed post-harvest sunflower seeds were immediately stored at −20 °C until the experiment. γ-ray irradiation was performed using a ^137^Csγ-ray irradiator (cat no. GM2000; Gamma-Service Medical, Leipzig, Germany). Sunflower seeds were sealed in polyethylene bags (100 g/bag) and treated as follows: 0, 2.0, 4.0, 5.0, 6.0, or 8.0 kGy.

#### 2.5.2. Post-Irradiation Mildew Incidence Rate in High-Humidity Conditions

Artificially accelerated mildew tests were used to compare internal mildew incidence rates under a high humidity: 100 sunflower seeds per irradiation dose were sealed in a desiccator with a KCl-saturated solution (RH 84%) and incubated at 28 °C in the dark for 30 days. Three replicates were performed per dose. After 30 days of incubation, the seeds were dehulled. Kernels with mildew spots, mycelium, or discoloration were counted as moldy, and the mildew incidence rate was calculated as the percent of moldy kernels. The results were compared using the TUKEY method with Proc ANOVA in SAS 9.1 (SAS Institute Inc., Cary, NC, USA).

#### 2.5.3. Acid and Peroxide Values of the γ-Irradiated Kernels

After irradiation, seeds were allowed to sit at natural storage temperatures for six months. The acid and peroxide values of the kernels immediately after irradiation and after six months of storage were measured using titration and colorimetry methods, respectively, as set forth in GB/T 5009.37-2003 [29]. The results were compared using the TUKEY method with Proc ANOVA in SAS 9.1.

## 3. Results

### 3.1. Effects of Mildew on the Fungal Community of the Sunflower-Seed Kernel

**Figure 1:**
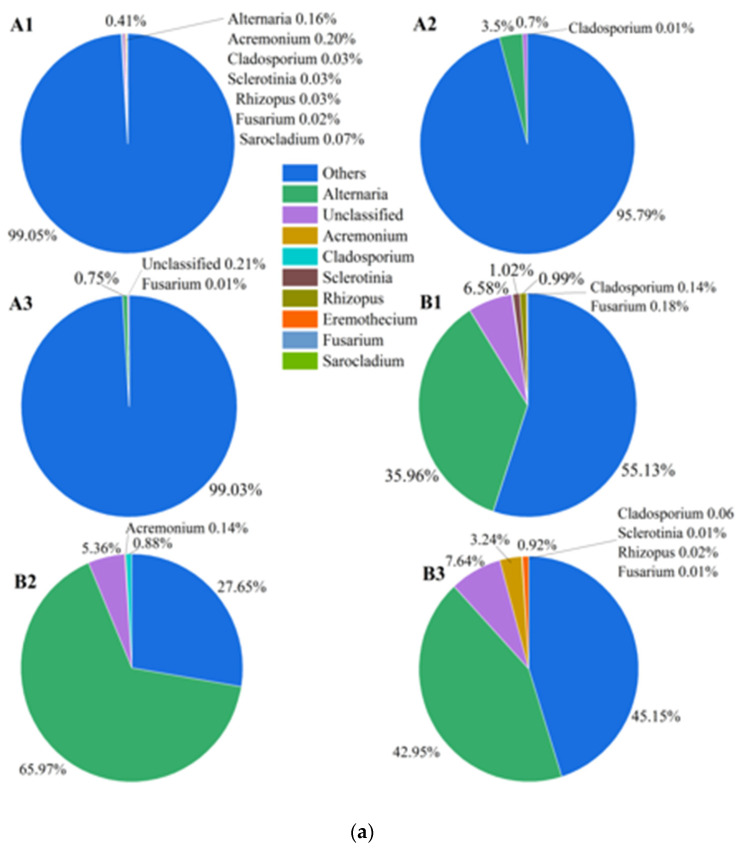

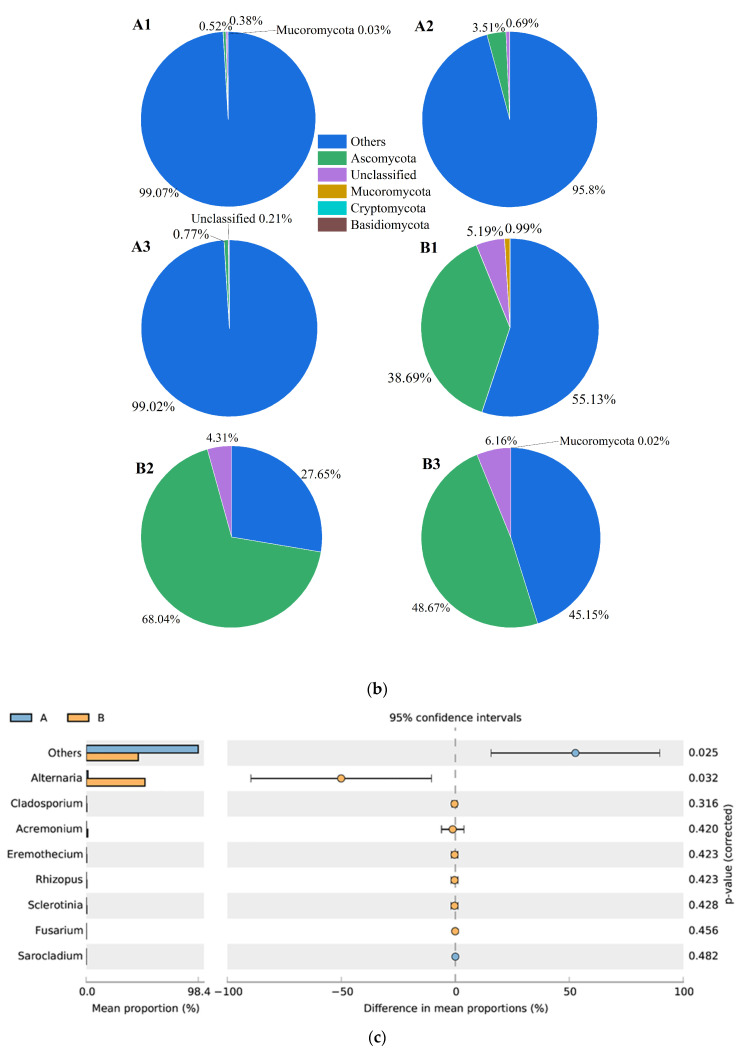
Differences in the composition and pollution degree in post-storage internally mildewed and normal fungus–seed symbiosis across three seasons. Group A (A1–A3), unmildewed (2018–2020); group B (B1–B3), mildewed (2018–2020). (**a**) Genus-level community composition; (**b**) phylum-level community composition; (**c**) STAMP analysis of groups A and B.

**Figure 2:**
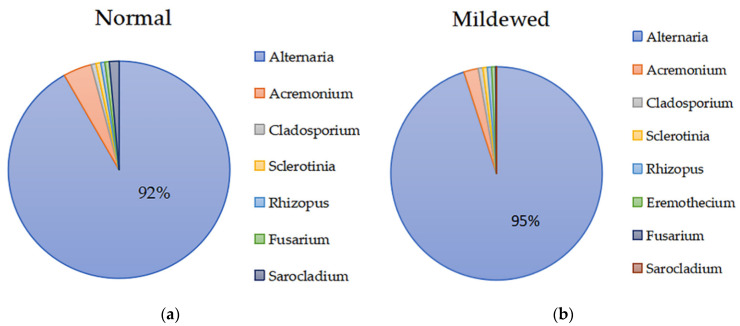
Fungal community structure in post-storage mildewed and unmildewed sunflower seed kernels. (**a**) Normal; (**b**) Mildewed. Note: Percentage was the mean value over three years (2018–2020).

A total of 230,226 sequence reads, with a median length 250 bp were obtained across all post-storage samples. The sequences were clustered into 58 OTUs using a 97% similarity cut-off. The normal and mildewed samples were assigned to group A and group B, respectively. For fungus–seed symbionts, the degree of microbial pollution in group B was much greater than that of group A due to the higher relative abundance of microbial OTUs (Figure 1a,b), consistent with expectations. STAMP analysis also identified significant differences between normal and mildewed fungus–seed communities. The abundance of the plant host was significantly lower in group B as compared to group A (*p* < 0.05), while the abundance of *Alternaria* was significantly greater in group A as compared to group B (*p* < 0.05) (Figure 1c). However, differences in the abundances of other genera were not significant. For fungal communities, the sequences from the samples represented a total of eight fungal genera and five fungal phyla. Almost all of the fungal genera identified were associated with field infections (*Alternaria*, *Acremonium*, *Rhizopus*, *Cladosporium*, *Fusarium*, *Sclerotinia*, and *Sarocladium*) (Figure 2). *Alternaria* dominated all samples in both group A (92.00%) and group B (95.00%) (Figure 2). The relative abundances of all other genera were low. The FUNGuild database was used to classify all identified fungi. The FUNGuild results showed that all the fungi except *Sarocladium* (saprophytic fungus) were plant pathogens, indicating that pathologic nutrition was an important characteristic of internally mildewed sunflower seeds.

**Figure 3:**
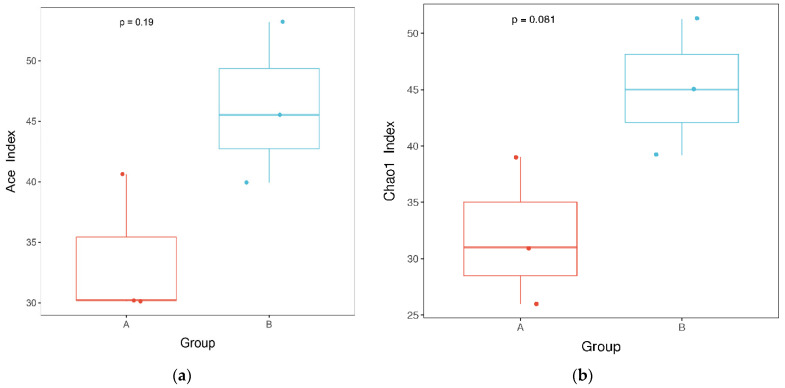
α-diversity of species communities in normal and mildewed post-storage fungus–seed symbiosis. (**a**) Ace index; (**b**) Chao index. Group A: unmildewed. Group B, mildewed.

The species richness was higher in the mildewed kernels than the normal ones, nevertheless, these differences were not significant (Figure 3). Similarly, for the fungal community structure, ANOSIM analysis identified no significant differences between the mildewed and normal samples (R = 0.704, *p* = 0. 1) (Supplementary Figure S1).

**Figure 4:**
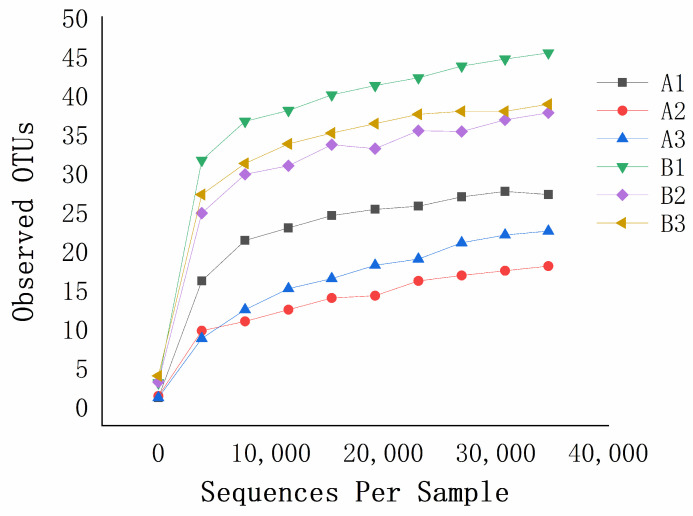
Rarefaction curves for the sunflower seed kernels. A1–A3, unmildewed (2018–2020); B1–B3, mildewed (2018–2020).

Rarefaction curves showed that the number of observed OTUs increased exponentially as the number of sequences increased, but then tended to plateau (Figure 4). These curves indicated that the sequences sufficiently described the fungal compositions of each sample.

### 3.2. Identification of the Pathogen Causing Mildew

**Figure 5:**
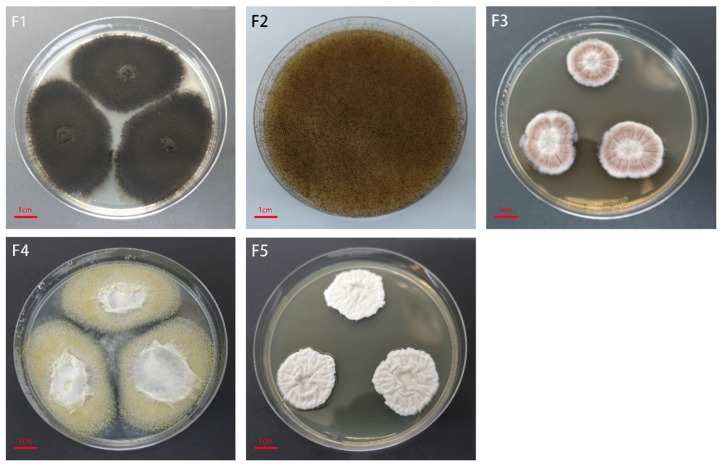
Characteristics of fungal colonies isolated from internally mildewed sunflower seeds. F1, *Alternaria* spp. F2, *Rhizopus stolonifera*. F3, *Aspergillus sydowii*. F4, *Aspergillus flavus*. F5, *Aspergillus niveus*.

After the culture of internally mildewed seed kernels, we isolated five single fungal colonies (Figure 5). Based on colony morphology and ITS sequence characteristics, the five isolates were identified as *Alternaria* spp., *Rhizopus stolonifer*, *Aspergillus sydowii*, *Aspergillus flavus*, and *Aspergillus niveus* (Table 1). Consistent with the sequencing results, *Alternaria* spp. was the dominant taxa and the incidence of *Aspergillus* spp. was extremely low. Interestingly, although the relative abundance of *Rhizopus* spp. was low, *Rhizopus* colonies grew very large, spreading over the plate due to the sprawling mycelium of this taxon. This also demonstrated that the plate culture method does not accurately reflect microbial abundance.

**Figure 6:**
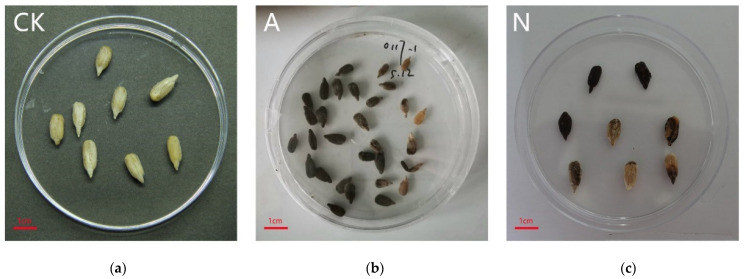
Sunflower seed kernels after reinoculation with mildew-inducing fungi. (CK, sterile water; A, *Alternaria* spp.; N, post-storage kernels with naturally occurring internal mildew.). (**a**) CK; (**b**) A; (**c**) N.

Sterile, unmildewed kernels were inoculated with the dominant taxa of *Alternaria* spp. After seven days of culture, the kernels inoculated with *Alternaria* spp. (an isolate with a dark brown phenotype) were obviously mildewed, with dark spots or large dark patches. In contrast, the blank control remained unmildewed (Figure 6a). Mildew phenotypes were similar between the naturally mildewed seed kernels (Figure 6c) and the sterile seed kernels inoculated with *Alternaria* spp. (Figure 6b), suggesting that naturally mildewed seeds were also infected with *Alternaria* spp.

### 3.3. Investigation of the Source of Internal Mildewing during Field-Planting and Storage Stages

#### 3.3.1. Sunflower Seeds Are the Most Vulnerable to Internal Mildew Naturally during Field-Planting

**Figure 7:**
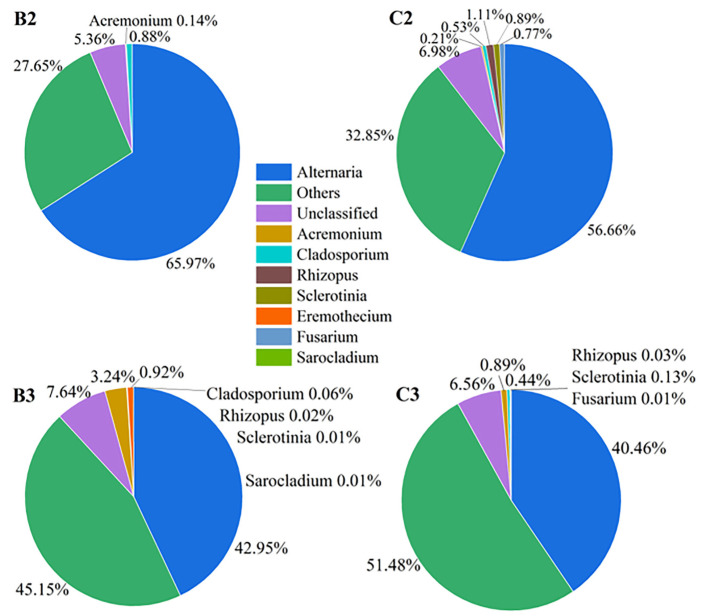
Genus-level community composition of internally mildewed sunflower seed kernels. (B2,B3), post-storage seeds (2019–2020). (C2,C3), post-harvest seeds (2019–2020).

**Figure 8:**
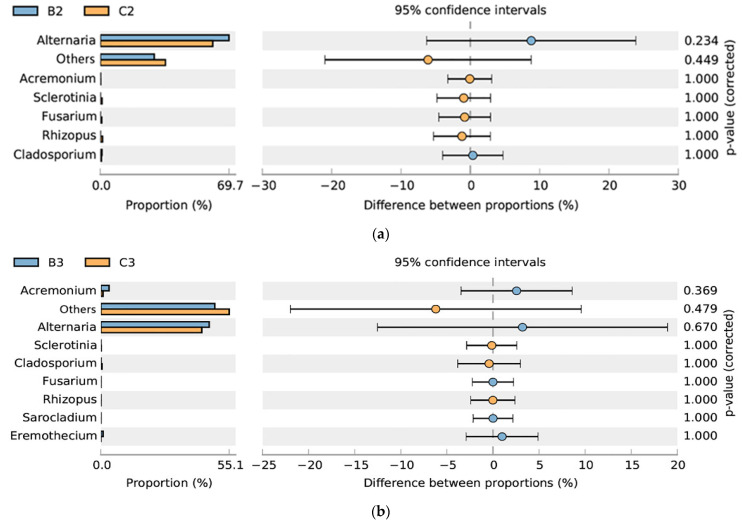
STAMP analysis of internally mildewed fungus–seed symbiosis. B, post-storage seeds. C, post-harvest seeds. (**a**) B2, B3, post-storage seeds (2019–2020); (**b**) C2, C3, post-harvest seeds (2019–2020).

Post-storage mildewed samples and post-harvest mildewed samples were assigned respectively to group B and group C, respectively (Figure 7). The relative abundances of all symbiotic seed–fungal OTUs were similar between group B and group C, with no significant differences (Figure 8a,b). Fungal community composition between the post-storage and post-harvest mildewed samples was also similar, with no significance (Supplementary Figure S2).

#### 3.3.2. The Conditions for Occurrence of Internal Mildew during Storage

**Figure 9:**
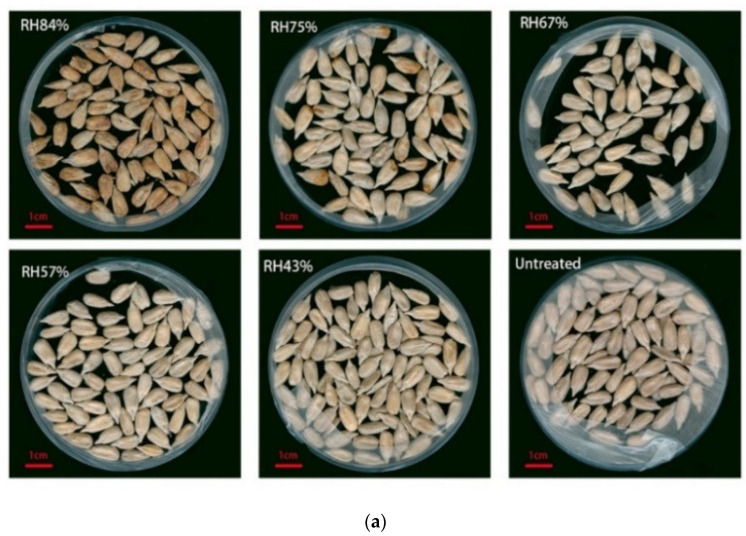

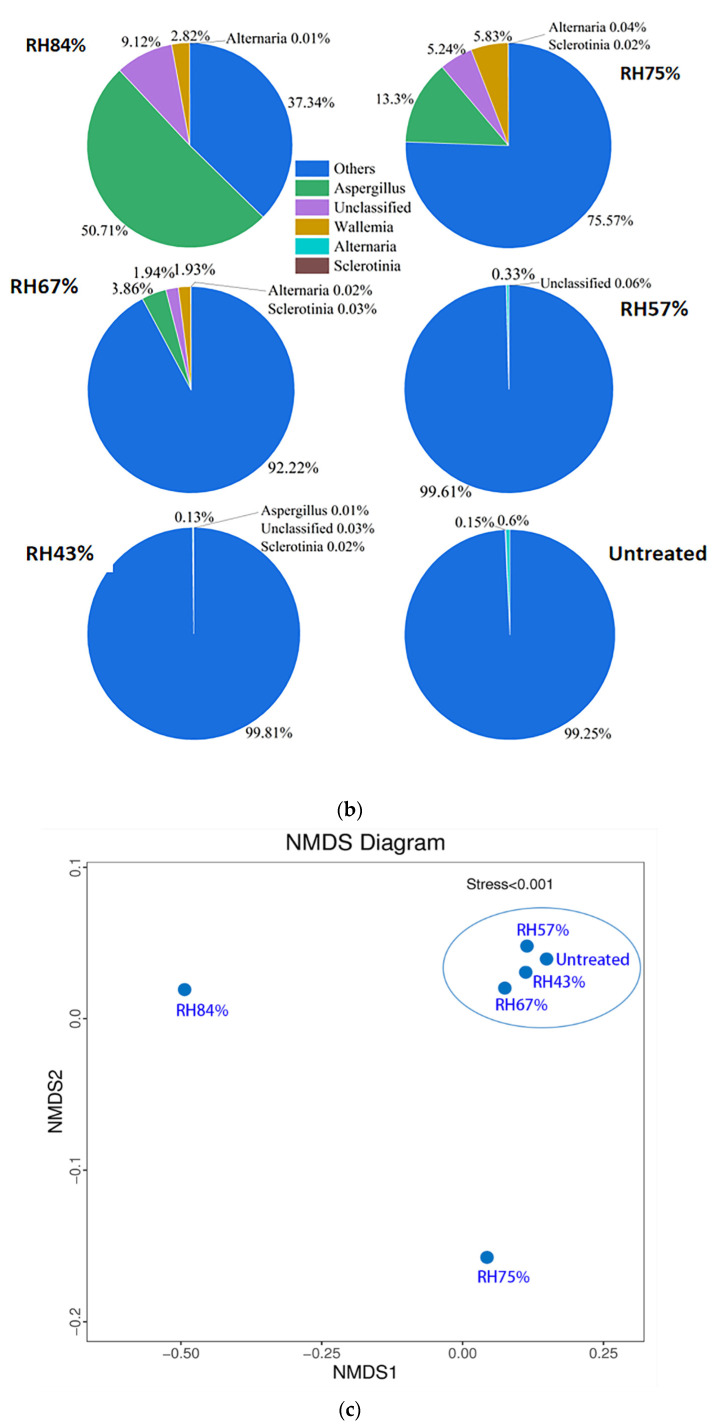
The effects on unmildewed sunflower seed kernels exposed to various levels of relative humidity (RH) for six months. (Untreated: unmildewed post-harvest kernels) (**a**) Kernel appearance; (**b**) Genus-level microbial structure; (**c**) NMDS analysis showing similarity among fungal populations from kernels of sunflower seeds exposed to various levels of relative humidity (RH) for six months.

The initial moisture content of the sunflower seed kernels was 5.2%. After six months at RHs of 84, 75, 67, 57, and 43%, the moisture contents of the kernels were 8.81, 7.72, 5.6, 5.07, and 3.98%, respectively (Table 2). Consistent with expectations, the degree of microbial pollution in the samples increased concomitantly with increasing RH. However, for the appearance, mildew was not visually obvious until the RH was greater than 75% (Figure 9a). At RHs less than 67%, the sunflower seed kernels remained mold-free, and *Alternaria* dominated the microbial community (Figure 9b). When RH reached 67%, *Aspergillus* became the dominant fungal genus despite an absence of obvious moldy visual signs. As RH continued to increase, the relative abundance of *Aspergillus* increased concomitantly, and signs of mildew appeared and grew more severe (Figure 9a,b). Interestingly, the relative abundances of *Alternaria* and other field fungi were low both at a RH < 67% and at a RH ≥ 67% (Figure 9b). NMDS analysis indicated that the microbial communities of the samples exposed to lower humidities (RH < 67%) and the unmildewed pre-storage kernels were more similar to each other than to the samples exposed to higher humidities (RHs ≥ 75%); the populations associated with samples exposed to 75% RH and 84% RH were also highly dissimilar (stress ≤ 0.2; Figure 9c). The NMDS results and microbial changes were consistent with the kernel appearance, suggesting that 67% may be a critical RH threshold. 

#### 3.3.3. Irradiation Treatment to Prevent the Occurrence of Internal Mildew during Storage under High-Humidity Conditions

**Figure 10:**
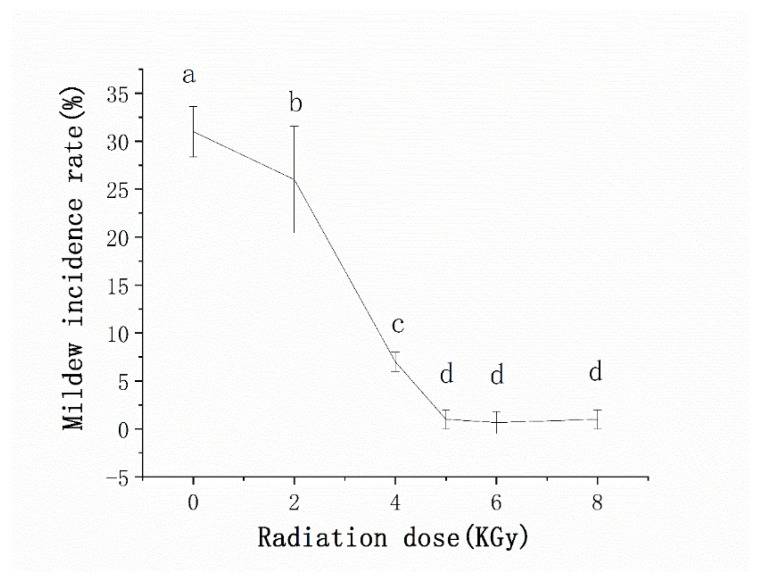
Mildew incidence rate from unmildewed sunflower seeds after various doses of γ radiation. Means with different lowercase letters are significantly different (*p* < 0.05). Bars represent the standard deviations.

**Figure 11:**
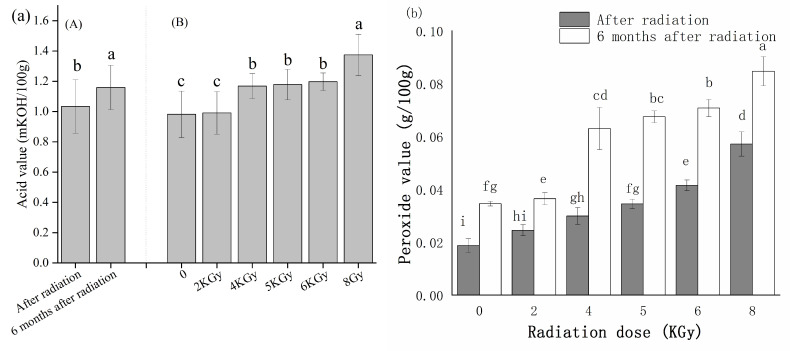
The edible quality of unmildewed sunflower seeds after various doses of γ radiation. Means with different lowercase letters are significantly different (*p* < 0.05). Bars represent the standard deviations. (**a**) Acid values [(A) Storage time; (B) Radiation dose.]; (**b**) peroxide values.

The artificially accelerated mildew test showed that treatment with at least 5 kGy γ-ray irradiation significantly inhibited mildew growth in high humidity (Figure 10). However, it is important to note that the radiation dose should not be increased with impunity. We found that both the acid value and peroxide value increased significantly (*p* < 0.05) in the irradiated seeds as compared to the unirradiated seeds, both immediately following treatment and after six months of storage (Figure 11a,b). The interaction between radiation dose and storage time did not have a significant effect on acid value (*p* > 0.05), and thus only the main effects were compared (Figure 11a). Interestingly, the peroxide value in sunflower seed kernels treated with 4 kGy increased suddenly after six months of storage, but peroxide values immediately following treatment only increased slightly as radiation dose increased (Figure 11b). This suggested that storage duration affected the peroxide value. However, even at 8kGy irradiation, peroxide and acid levels in the irradiated kernels were well below 0.38 g/100 g and 4 mg KOH/100 g, respectively, which are the maximum levels specified in the NY/T 902-2004 [30]. Specifically, immediately after irradiation at 5 kGy, after six months of storage at natural storage temperature, the peroxide content was 0.0675 g/100 g and the acid content was 1.2157 mg KOH/100 g (Figure 11a,b).

## 4. Discussion

### 4.1. Alternaria Was the Only Dominant Pathogenic Fungus Causing the Internal Mildewing of Post-Storage Sunflower-Seed Kernels

Previous studies have investigated the microbial compositions of normal sunflower seeds. In South American and European countries, *Alternaria* spp. and *Fusarium* spp. dominate in normal sunflower seed kernels [31]. A recent study assessed the fungal community of the normal Chinese sunflower seed kernels using the plate culture method and showed that the dominant fungal taxon was *Alternaria* spp., followed by *Fusarium* spp. and *Penicillium* spp. [12]. However, few studies have assessed the relationship between microbial compositions and internal mildewing. In China, internal mildewing is generally believed to be associated with *Aspergillus flavus* due to its strong carcinogenicity [32], although there are currently insufficient data to support this claim. Therefore, the first aim of this study was to investigate the influence of internal mildewing on the fungal community of sunflower seed kernels and the mildew-causing pathogenic fungi. 

To date, there have been several reports on the mildewing of hull-less crops but few on the internal mildewing of crops with hulls [33]. Previous research on tobacco leaves demonstrated the Chao index was significantly lower in mildewed tobacco leaves as compared to healthy ones [34]. In addition, Welty and Lucas [35] found mildew changed the composition of fungal communities in tobacco leaves: *Pyrenophora*, *Alternaria*, *Radulidium*, and *Cryptococcus* were identified in healthy tobacco leaves, while the most dominant fungi in the mildewed leaves were *Aspergillus* spp. and *Penicillium* spp.

In the present study, internal mildewing did not significantly alter the composition of the fungal community of sunflower seed kernels (Supplementary Figure S1). *Alternaria* was by far the dominant taxon, with all other fungi present at very low levels in both normal and mildewed kernels (Figure 2). Thus, the fungi carried by moldy kernels are already present in normal kernels, with the contamination degree of mildew-causing fungi insufficient to cause visible mildew, as shown in Figure 1. The fungal community of normal sunflower seeds found herein was consistent with a previous study [12] and explains the high content of *Alternaria* toxin in normal sunflower seeds by European Food Safety Authority [36]. Similar to the lack of change in fungal community, the richness also did not change significantly after internal mildewing (Figure 3). We speculate that the hull may protect the sunflower seed kernels from contamination by external microorganisms, reducing the effects of mildewing on the microbial community composition in comparison to hull-less crops. In addition, *Alternaria* is the only dominant mildew-causing pathogen. The ability of fungal species to colonize and occupy specific ecological niches depends on their ability to effectively compete with other microorganisms. Thus, we speculate that *Alternaria* (plant pathogen) may be better at exchanging the resources with host cells by damaging them for reproduction due to the highly nutrition-selective nature, than other plant pathogens. 

The *Alternaria* strain isolated from the moldy kernels was identified as the only abundant mildew-causing fungus in sunflower-seed kernels based on morphological characters, molecular characteristics and re-inoculation. Consistent with this, STAMP analysis revealed that *Alternaria* was the only fungal taxon that was significantly more abundant in the mildewed seed–fungus symbiosis community as compared to the unmildewed community (Figure 1c), reflecting its high correlation with internal mildewing. Previous researches have shown that *Alternaria* infections can result in leaf wilting, leaf spot, and stem spot diseases in sunflower [37], but few studies have investigated *Alternaria* infections in seeds. However, sunflower seeds deserve special attention for *Alternaria* spp. and the corresponding mycotoxins can enter the human food chain with the seeds. In recent years, *Alternaria* spp. have received extensive attention from government authorities and researchers as emerging mycotoxins. At present, *Alternaria* toxins are considered potentially harmful food contaminants, due to the incompleteness of available data [38]. However, a long-term survey by the European Food Safety Authority showed that sunflower seeds are highly susceptible to have higher contents of *Alternaria* toxin content in comparison to other oil crops [39]. Therefore, it is necessary to control *Alternaria* spp. infections in sunflower seeds, although the potential dietary risk of exposure to *Alternaria* spp. may be lower than that of storage-associated mycotoxins (i.e., aflatoxin).

### 4.2. Sunflower Seeds Are Most Vulnerable to Internal Mildew during Field-Planting

To date, almost all studies of microorganisms and their ability to colonize sunflower seeds have been based on sunflower seeds at time of sale [12,13]. To our knowledge, no previous studies have investigated microbial changes during the sunflower seed production cycle. Therefore, one of the main objectives of this work was to explore the possible sources of internal mildewing throughout the sunflower-seed production cycle.

According to industry norms [40], sunflower seeds are field planted, threshed after harvest, stored in normal-temperature warehouses typically for about 6–8 months, transported to different sales regions; and processed before sale in China. Sunflower seed kernels can be contaminated by microorganisms at any stage between planting and processing. However, internal mildew rarely occurs during transportation and processing because these stages are too brief for mildew to develop. Therefore, we assessed post-harvest and post-storage sunflower seeds in present study. In the naturally mildewed samples, the fungal composition and mildewing degree were similar between the mildewed seeds post-harvest and post-storage (Figure 7 and Figure 8). This suggested that sunflower seeds are the most vulnerable to internal mildewing during the field-planting stage due to the multiplication of *Alternaria*, and there is no significant development of internal mildewing during the storage stage. We speculated that *Alternaria* multiplication might take place late in seed development, as the appearance of the internally moldy sunflower seeds did not differ noticeably from that of normal seeds. Indeed, Krishnappa, M. et al. [41] proposed that *Alternaria* reproduction at an early stage of seed development is likely to lead to more serious damage to seeds than internal mildewing. In Inner Mongolia, sunflower seeds are usually harvested in September–October, and the annual rainfall is concentrated in July–August. This climatic pattern creates suitable conditions for the reproduction of *Alternaria* and the occurrence of internal mildewing late in seed development. Consistent with this, the multiplication of field fungi under the highly humid conditions (RH 90–100%) have been previously reported; humid conditions occasionally occur in southwestern China, leading to field fungi outbreaks in fields before the soybean harvest [42]. Furthermore, our study showed that *Alternaria* was dominant in normal sunflower seeds, suggesting that *Alternaria* is seedborne. Consistent with this possibility, *Alternaria* was shown to be the dominant seedborne taxon in Chinese sunflower seed kernels [12]. Indeed, *Alternaria* spp. was shown to be seedborne and to overwinter in diseased sunflower residues [37]. In addition, *Alternaria* can travel through the plant tissues from the seed itself and colonize the sunflower seed kernel both externally and internally [16]. Thus, we speculated that *Alternaria* originated from seed transmission and colonized at the seed-development stage. Finally, only a few seeds developed internal mildew, with *Alternaria* contamination only reaching necessary levels under humid field conditions. 

Previous studies have shown the correlations between the indoor and outdoor climate [43]. Thus, the outdoor climate determines the microclimate of the sunflower seeds. According to industry norms, sunflower seeds are usually stored in normal-temperature warehouses. However, the storage RH can be regulated to some extent using suitable measures. Thus, the critical RH threshold was studied under natural storage temperatures in present study. NMDS analysis showed that climatic factors play important roles in shaping microbial changes during storage, with 67% being the critical threshold in normal-temperature warehouses. After an average storage duration (six months), *Aspergillus* spp. occupied the dominant ecological niche at RHs greater than 67% (moisture content = 5.6%). Consistent with this, Robertson J.A. [44] showed that a moisture content ≥6% was required for *Aspergillus* invasion, although invasion was very low when the moisture content was <6.5%. Identical with these microbial changes, internal mildewing also happens at RHs greater than 67%.

Notably, internal mildew never occurred at conditions below the RH threshold under normal-humidity storage conditions (RH 40–67% under natural storage temperatures); the microbial community hardly changed over the six months. Throughout this study, the humidity of the warehouse was recorded during storage, and all RH values were below 65%. Thus, in most cases, sunflower seeds will remain mold-free during storage in the primary sunflower-seed production areas. Interestingly, the relative abundances of *Alternaria* and other field fungi were low both at a RH < 67% and at a RH ≥ 67%, suggesting that *Alternaria* and other field fungi that had colonized the kernels during the field-planting stage cannot multiply to exacerbate mildewing during storage (even under high-humidity storage condition). In addition to climatic factors, biological factors should not be ignored. However, our 18S results did not indicate that another biological factor was a force driving microbial change and internal mildewing. 

### 4.3. A Combination of Field Management Targeting Alternaria and 5 kGy γ-ray Radiation Can Control Internal Mildew

Based on our results, appropriate methods can be established to control the growth of mildew-producing fungi. Because internal mildewing mainly occurs during the field-planting stage, field management against *Alternaria* should be the preferred direction. Previous studies have shown that the indiscriminate use of synthetic antifungals can lead to the development of resistant strains, requiring higher doses of fungicides, with the consequent increase in toxic residues in food products. Recently, Avenot and Michailides [45] found that *Alternaria* strains collected from commercial pistachio orchards had developed resistance to two fungicides applied in the field, cyprodinil and fludioxonil. However, some plant extracts and anti-*Alternaria* microorganisms have proved effective in reducing *Alternaria* spoilage in fruits and vegetables; these could be adopted as an alternative to synthetic agents [46,47]. *Alternaria* spp. was also shown to overwinter in diseased sunflower residues [37]. Thus, field managements techniques, such as crop rotation, deep ploughing and soil improvements, will also effectively reduce *Alternaria* spp. contamination. Therefore, a combination of natural substances and agricultural practices should be explored for the control of internal mildewing.

Although internal mildewing occurs less frequently during storage than field-planting, storage-associated fungi must be prevented, as short-term exposure to a high humidity can rapidly increase storage-mildew growth. Therefore, environmental dehumidification is essential. In addition, γ-ray irradiation is a powerful measure to prevent mildew during storage. To date, few studies have investigated γ-rays for the anti-fungal treatment of sunflower seeds before storage. However, Darfour, B. et al. showed that 2.5–10 kGy γ-ray irradiation did not impair the edible quality and nutritional content of the cola nut [48], while Song found that 5 kGy γ-ray irradiation reduced three pathogens on pistachios to the detection limit (10 log CFU/g) without any change in appearance [49]. Similar to these previous studies of nuts, our results suggested that 5 kGy γ-ray irradiation can significantly reduce the incidence of mildew under high-humidity storage conditions without impacting edible quality after storage. 

## 5. Conclusions

Sunflower seeds are susceptible to internal mildew contamination. In this work, a comprehensive study was performed to study the source of internal mildewing across the sunflower seed production cycle. The results showed that *Alternaria* was the predominant mildew-causing pathogen of sunflower seeds for consumption. The results also indicated that sunflower seeds are most vulnerable to internal mildew, infected with *Alternaria* spp. at the field-planting stage. Additionally, the microbial changes related to internal mildewing were mainly determined by climatic factors. However, the application of anti-*Alternaria* practices, in conjunction with pretreatment with 5 KGy γ-irradiation and drying at the time of storage, will minimize or even avoid internal mildew contamination, further avoid mycotoxin entering the food chain. 

## Figures and Tables

**Table 1:** Molecularly identified fungal species in the mildewed sunflower seeds.

No.	Taxon	Percent Identity	Accession
F1	*Alternaria* sp.	99.45%	MK649978.1
F2	*Rhizopus stolonifer*	100%	MK722197.1
F3	*Aspergillus sydowii*	99.45%	MH625698.1
F4	*Aspergillus flavus*	99.65%	MW757217.1
F5	*Aspergillus niveus*	100%	MK590295.1

**Table 2:** Moisture contents and appearances of sunflower seed kernels incubated at various relative humidities (RHs).

Equilibrated RH (%)	Moisture Content (%)	Appearance
84	8.81%	Wrinkled, dark-brown spots
75	7.72%	Yellow spots
67	5.6%	Normal
57	5.07%	Normal
43	3.98%	Normal

## Data Availability

The data during the current study are available from the corresponding author on reasonable request.

## Supplementary Materials

The following supporting information can be downloaded at: https://www.mdpi.com/article/10.3390/microorganisms10071434/s1, Figure S1: ANOSIM and STAMP analysis of fungal communities in normal and mildewed post-storage sunflower-seed kernels; Figure S2: STAMP analysis of fungal community compositions in internally mildewed sunflower-seed kernels.
