# Supplementary material for: Characteristics of Fungal Communities and Internal Mildew Occurrence during the Stages of Planting and Storing of Sunflower Seed in China
Source: Microorganisms. 2022 Jul 15;10(7):1434. doi: 10.3390/microorganisms10071434 (PMC9318822; doi:10.3390/microorganisms10071434)
Supplement: Supplementary file 1 [file microorganisms-10-01434-s001.zip › microorganisms-1807879-supplementary.pdf]

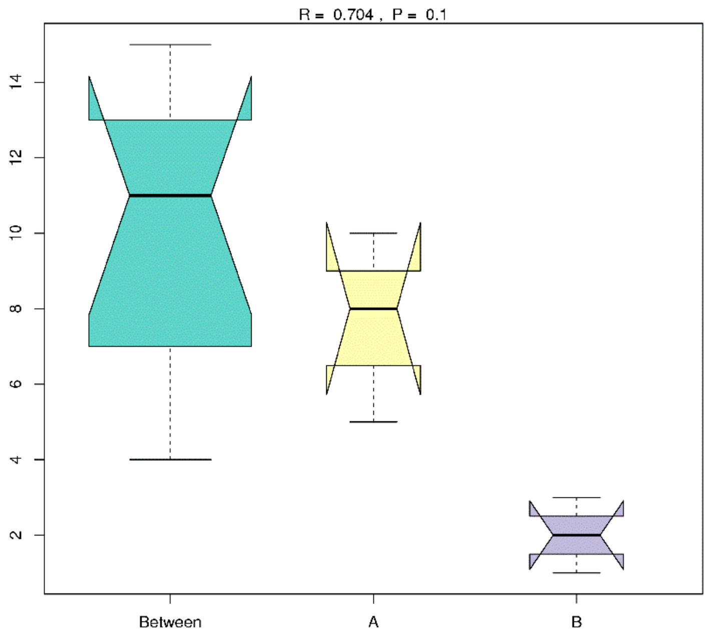

(a)

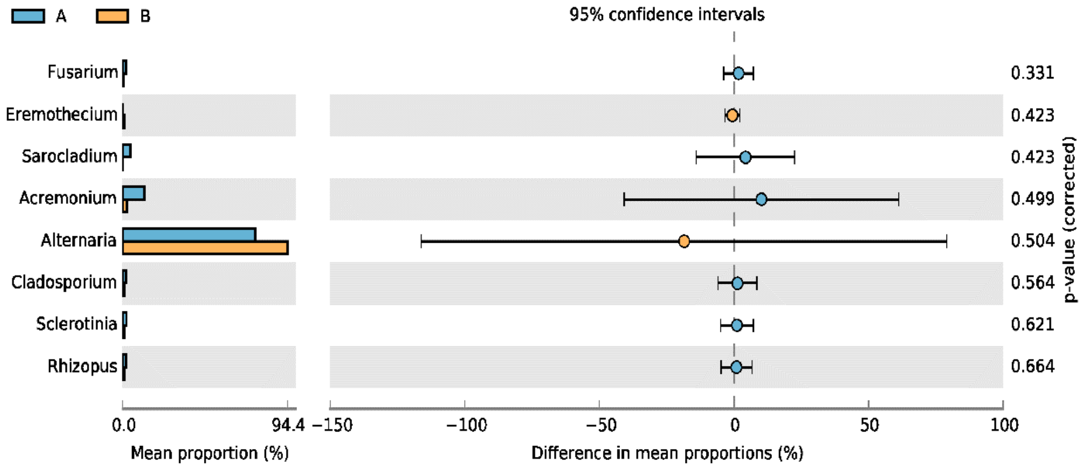

(b)

Supplement Figure S1. ANOSIM and STAMP analysis of fungal communities in normal and mildewed post-storage sunflower-seed kernels. Group A: unmildewed. Group B, mildewed. (a) ANOSIM. (b) STAMP

ANOSIM and STAMP analysis identified no significant differences between the mildewed and normal samples (R = 0.704, p = 0. 1), suggesting that the fungal community did not change significantly after internal mildewing.

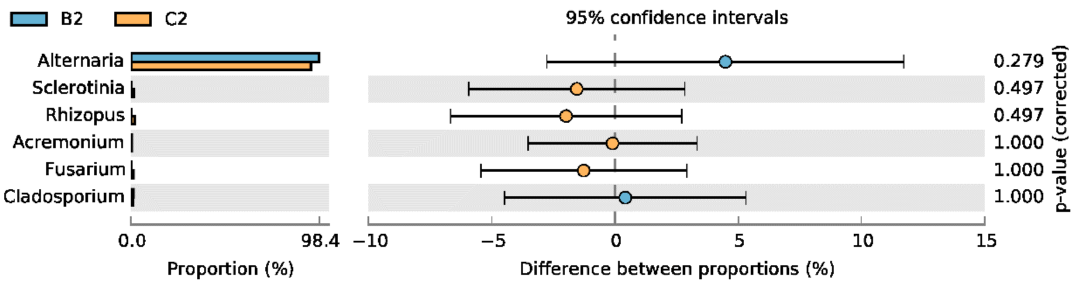

(a)

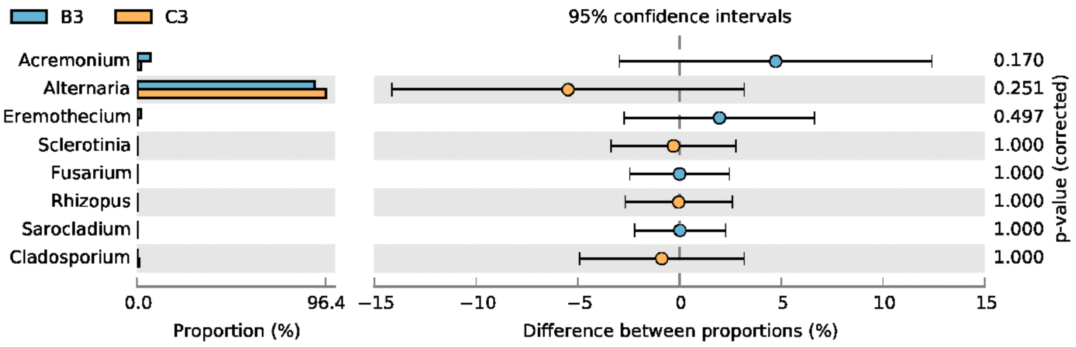

(b)

Supplement Figure S2. STAMP analysis of fungal community compositions in internally mildewed sunflower-seed kernels. B, post-storage seeds. C, post-harvest seeds. (a) B2, B3, post-storage seeds (2019-2020). (b) C2, C3, post-harvest seeds (2019-2020).

Fungal community composition between the post-storage and post-harvest mildewed samples was also similar, with no significances.
